# Comparative analysis of human microglial models for studies of HIV replication and pathogenesis

**DOI:** 10.1186/s12977-020-00544-y

**Published:** 2020-11-19

**Authors:** Mohammad A. Rai, Jason Hammonds, Mario Pujato, Christopher Mayhew, Krishna Roskin, Paul Spearman

**Affiliations:** 1 Division of Infectious Diseases, Cincinnati Children’s Hospital, 3333 Burnet Avenue, MLC 7017, Cincinnati, OH 45229 USA; 2 Division of Biomedical Informatics, Cincinnati Children’s Hospital, Cincinnati, OH USA; 3 Department of Pediatrics, University of Cincinnati School of Medicine, Cincinnati, OH USA; 4 Division of Infectious Diseases, Department of Medicine, University of Cincinnati School of Medicine, Cincinnati, OH USA; 5 Pluripotent Stem Cell Core Facility, Cincinnati Children’s Hospital, Cincinnati, OH USA

**Keywords:** Microglia, HIV-associated neurocognitive disorder, Induced pluripotent stem cell, HIV-1, Gene expression profiling

## Abstract

**Background:**

HIV associated neurocognitive disorders cause significant morbidity and mortality despite the advent of highly active antiretroviral therapy. A deeper understanding of fundamental mechanisms underlying HIV infection and pathogenesis in the central nervous system is warranted. Microglia are resident myeloid cells of the brain that are readily infected by HIV and may constitute a CNS reservoir. We evaluated two microglial model cell lines (C20, HMC3) and two sources of primary cell-derived microglia (monocyte-derived microglia [MMG] and induced pluripotent stem cell-derived microglia [iPSC-MG]) as potential model systems for studying HIV-microglia interactions.

**Results:**

All four microglial model cells expressed typical myeloid markers with the exception of low or absent CD45 and CD11b expression by C20 and HMC3, and all four expressed the microglia-specific markers P2RY12 and TMEM119. Marked differences were observed upon gene expression profiling, however, indicating that MMG and iPSC-MG cluster closely together with primary human microglial cells, while C20 and HMC3 were similar to each other but very different from primary microglia. Expression of HIV-relevant genes also revealed important differences, with iPSC-MG and MMG expressing relevant genes at levels more closely resembling primary microglia. iPSC-MG and MMG were readily infected with R5-tropic HIV, while C20 and HMC3 lack CD4 and require pseudotyping for infection. Despite many similarities, HIV replication dynamics and HIV-1 particle capture by Siglec-1 differed markedly between the MMG and iPSC-MG.

**Conclusions:**

MMG and iPSC-MG appear to be viable microglial models that are susceptible to HIV infection and bear more similarities to authentic microglia than two transformed microglia cell lines. The observed differences in HIV replication and particle capture between MMG and iPSC-MG warrant further study.

## Background

HIV-associated neurocognitive disorder (HAND) encompasses a broad range of neurocognitive dysfunction associated with HIV infection, including HIV-associated asymptomatic neurocognitive impairment (ANI), HIV-associated mild neurocognitive disorder (MND), and HIV-associated dementia (HAD) [1]. Antiretroviral therapy (ART) has been successful in dramatically reducing the incidence of HIV associated comorbidities, including HAD [2]. However, ANI and MND continue to be frequently encountered in clinical practice, even in individuals with undetectable viral loads [3, 4]. Estimates for the prevalence of HAND in persons living with HIV vary from 20% to as high as 50% [3, 5, 6]. There is no definitive laboratory test or specific therapy available for HAND. Multiple factors are likely to contribute to the development of HAND, including the residual effects of CNS damage in individual patients prior to starting ART, immune and glial cell activation, HIV-associated comorbidities, neurotoxicity from antiretrovirals, and the persistence of HIV replication in CNS tissues [7]. Given the high prevalence and significant morbidity attributed to HAND in the era of ART, this is an area deserving of intensive research efforts. The development of model systems that will inform the understanding of HAND are of significant priority in this effort.

HIV-1 infection of microglia was first described in 1986 in an autopsy study [8], and multiple studies have since reported HIV-1 RNA or protein expression in brain tissues [9–13]. HIV reaches the brain early in the course of infection, most likely through infected lymphocytes or myeloid cells, and infects perivascular macrophages and microglia [7, 14, 15]. Astrocytes have also been shown to be infected, although their role remains controversial [16, 17]. Nonhuman primate models of SIV infection have clearly shown that infection of the CNS occurs soon after primary viremia [18], and indicate that microglia are infected and may serve as a viral reservoir [19]. Analysis of viral isolates derived from the CNS reveal compartmentalized replication of CCR5-tropic isolates that are adapted to replication in macrophages/microglia [14, 20]. CNS macrophages/microglia are the likely source of some episodes of viral escape occurring after years of suppressive antiretroviral therapy [21]. Microglia are the resident tissue macrophages of the brain, derived from yolk sac progenitors, and are known to interact extensively with surrounding brain parenchymal cells including neurons and astrocytes, where they perform many essential functions [22–24]. In HIV-1 infection, microglia may contribute to CNS dysfunction and the development of HAND through excessive or unchecked activation [25] and may also serve as a viral reservoir [19, 26, 27]. Understanding HIV-1 interactions with microglia will likely be important in developing a comprehensive strategy to prevent or treat HAND.

One of the major limitations in studying HIV-microglia interactions is the limited availability of primary sources of human microglial cells, which can be derived from aborted fetal tissue or postmortem brain tissue. To overcome this limitation, renewable or continuous sources of microglia have been developed. The HMC3 line was established in 1995 through SV40-dependent immortalization of human embryonic microglial cells, and has been used extensively as a model for microglial cells [28, 29]. This cell line and subsequent derivatives have also been termed CHME-5 [30] and C13-NJ [31]. More recently, Alvarez-Carbonell and coworkers [32] transformed cells from adult brain tissue with lentiviral vectors expressing SV40 T antigen or a combination of SV40 T antigen and hTERT [26]. They demonstrated that these immortalized cells have microglia-like morphology and express key microglial surface markers, and utilized them for generating latently infected clones that reactivate HIV in response to inflammatory signals [32]. Another approach employed to derive authentic microglia involves differentiation of peripheral blood monocytes into microglia-like cells through applying specific combinations of chemokines and culture conditions [33–37]. These monocyte-derived microglia (MMG) have been demonstrated to have a phenotype and gene expression profile similar to human microglia, and have been used to model human neurodegenerative disease [37]. Rawat and Spector demonstrated that MMG were a viable model for studying HIV interactions with microglia, demonstrating that they were permissive and produced levels of virus comparable to primary human microglia [36].

In recent years technological advancements in induced pluripotent stem cell (iPSC) methodologies [38] have been applied for the generation of microglial cells [39]. The potential advantages to using iPSC methods includes the power to make abundant numbers of human-derived cells with an adult phenotype, and the ability to generate microglia expressing particular genetic characteristics or genetic alterations that facilitate mechanistic studies [39]. Similar to MMG protocols, there have been multiple methods described for deriving microglia from iPSCs [40–44]. Recently, the Blurton-Jones lab has published a greatly simplified protocol for generation of iPSC-derived microglia, and the cells derived have been shown to very closely resemble authentic microglia by phenotype, surface markers, and gene expression profiles [45].

We sought to evaluate these existing microglial cell models in order to determine the ideal model or models for studying HIV-microglial cell interactions. The ideal model would closely resemble authentic adult microglia in morphology, expression of prototypical microglial markers, and gene expression profile. Furthermore, we reasoned that the ideal model for studying HIV interactions should express HIV receptor and coreceptor proteins at levels similar to adult microglia, and should support HIV infection and replication. Finally, the microglial model should reproduce the levels of HIV restriction factors that are found in microglia from human brain. Here we highlight important differences between the immortalized C20 and HMC3 microglial cell models and two primary cell models, including significant differences in receptor and coreceptor expression and overall gene expression profiles. We found that iPSC-derived microglia most closely resemble adult microglia in gene expression, but cluster closely with MMG. The immortalized microglial cell lines, in contrast, were more distant from authentic microglia as indicated by gene expression studies, could not be infected without pseudotyping, and differed in restriction factor expression from authentic microglia. iPSC-MG and MMG were competent for HIV infection without the need for pseudotyping, and expressed levels of HIV restriction factors similar to that of authentic microglia. Despite their many similarities, there were differences in the magnitude and duration of HIV replication following HIV infection of iPSC-MG and MMG, and in the phenotype of HIV particle capture and retention by these microglia model cells.

## Results

### Derivation of iPSC-MG and MMGs and culture of C20, HMC3 cell lines

**Fig. 1:**
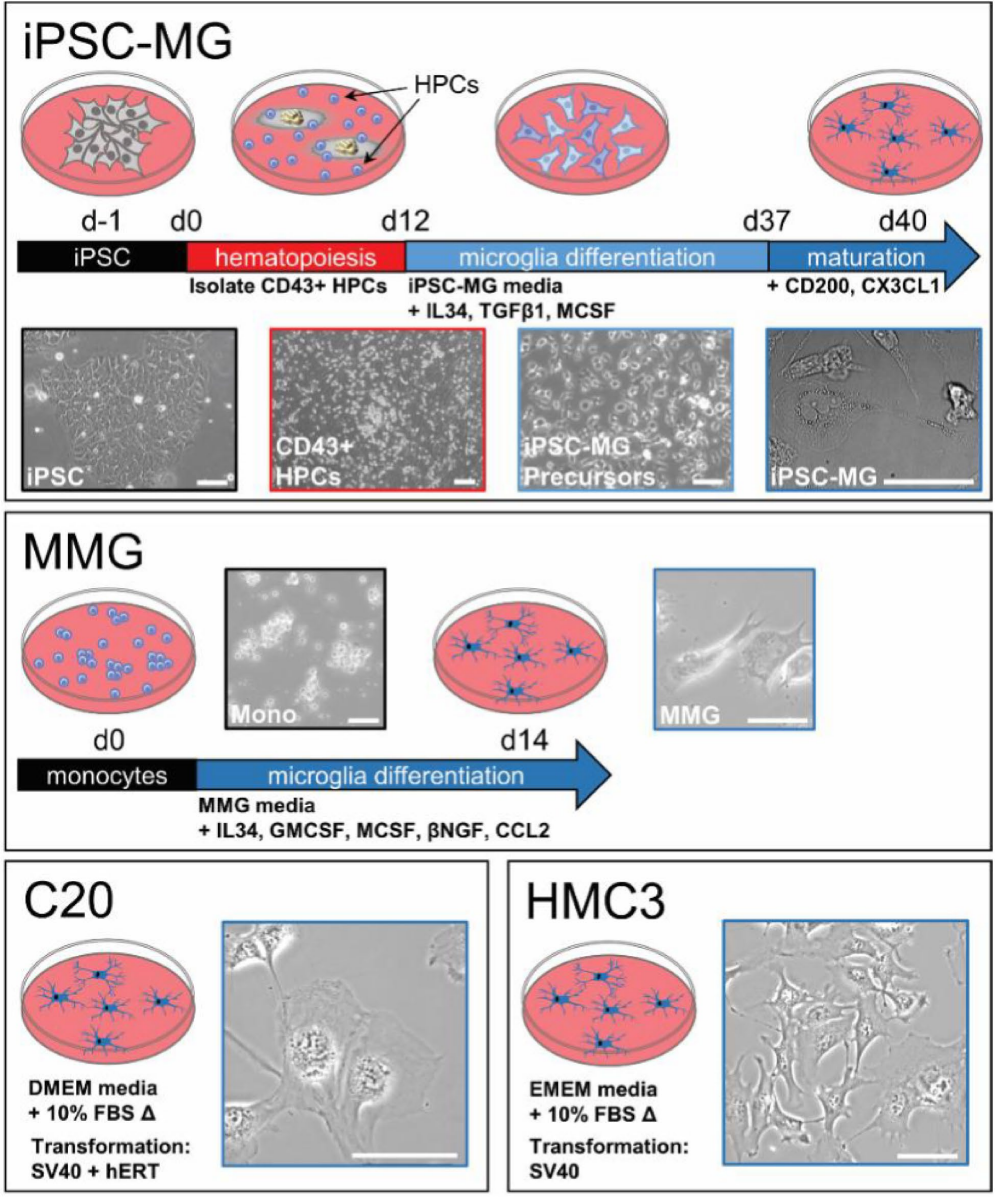
Schematic diagram illustrating the process and morphology of four different microglial cell types. Top panel: iPSCs progenitors undergo hematopoiesis (days 0–12) to form CD43+HPCs, then differentiate to form iPSC-MG Precursors (days 12–37), before finally maturing to form terminally differentiated iPSC-MG on day 40. Phase contrast images are shown below. Scale bar = 30 µm. Middle panel: Schematic showing differentiation of monocytes to MMG using a cytokine cocktail comprising IL-34, GM-CSF, M-CSF, Beta-NGF and CCL2. Scale bar = 18 µm. Bottom panel: Culture conditions for C20 and HMC3 cell types along with phase contrast images for these two cell types, scale bar = 25 µm

We utilized a protocol from the Blurton-Jones laboratory to develop iPSC-MG [45]. Briefly, iPSCs were first differentiated into CD43 + hematopoietic stem cells (CD 43 + HPCs) (Additional file 1: Fig. S1), followed by treatment with IL-34, TGF-β1, and M-CSF to generate iPSC-MG precursors, and subsequently a final maturation step with CD200 and CX3CL1-stimulation to produce iPSC-MG. Representative images of the cells at different timepoints in the differentiation process are shown in the top panel of Fig. 1. The resulting microglia exhibited an ovoid central body surrounding the nucleus and extended processes characteristic of these cells [43, 45]. Monocyte derived microglia (MMG) were generated according to the methods of Ryan et al. [37]. Monocytes were isolated from human PBMCs and cultured for fourteen days in media supplemented with IL-34, GM-CSF, M-CSF, B-NGF and CCL2 (Fig. 1, middle panel). The MMG displayed an oval central cell body and a ramified morphology after 10-14 days in culture (Fig. 1) [33, 34, 36]. C20 cells were originally generated from primary human microglia transformed using SV40 T antigen (SV40) and human Telomerase Reverse Transcriptase (hTERT), forming a clonal cell population [32]. These were obtained from the originators and were cultured according to their specifications [32]. C20 cells also demonstrated a ramified morphology as shown in Fig. 1, bottom left panel. The HMC3 cell line was originally generated through SV40-dependent immortalization of a human fetal brain-derived primary microglia culture [28, 29]. HMC3 cells were obtained from the American Type Culture Collection (ATCC) and cultured in the appropriate media, exhibiting multiple cellular ramifications as had been described (Fig. 1, bottom right panel) [29]. The four microglial cell models shown in Fig. 1 were chosen for further evaluation to determine their suitability for investigating HIV-microglia interactions.

### Expression of microglia-specific markers

**Fig. 2:**
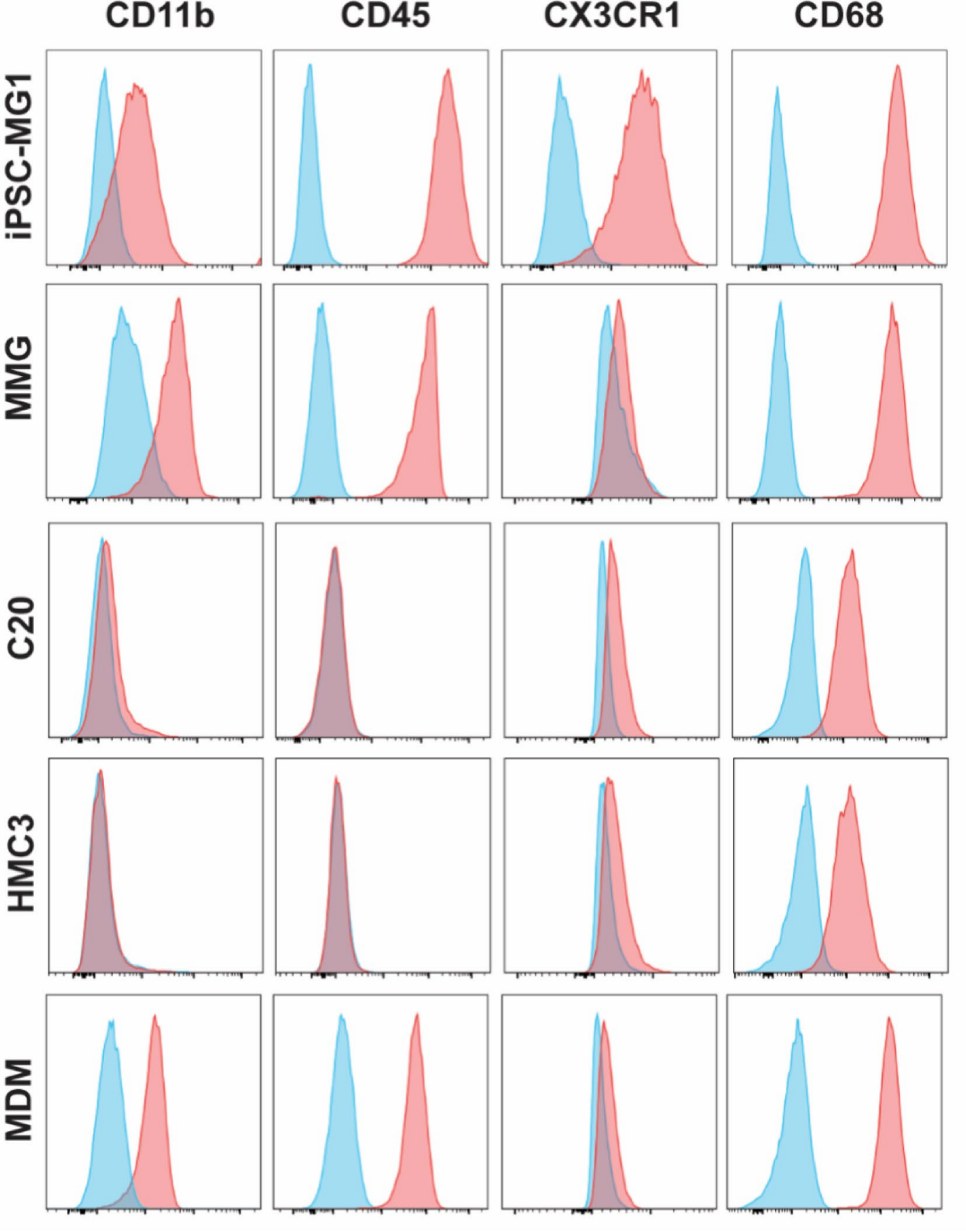
Flow Panel characterizing CD11b, CD45, CX3CR1, CD68, CD317 and CD169 for the different microglial cell types and MDMs as labelled on the left. Blue histogram represents isotype-control antibodies, red histograms represent the specific staining for markers indicated at the top of the figure

**Fig. 3:**
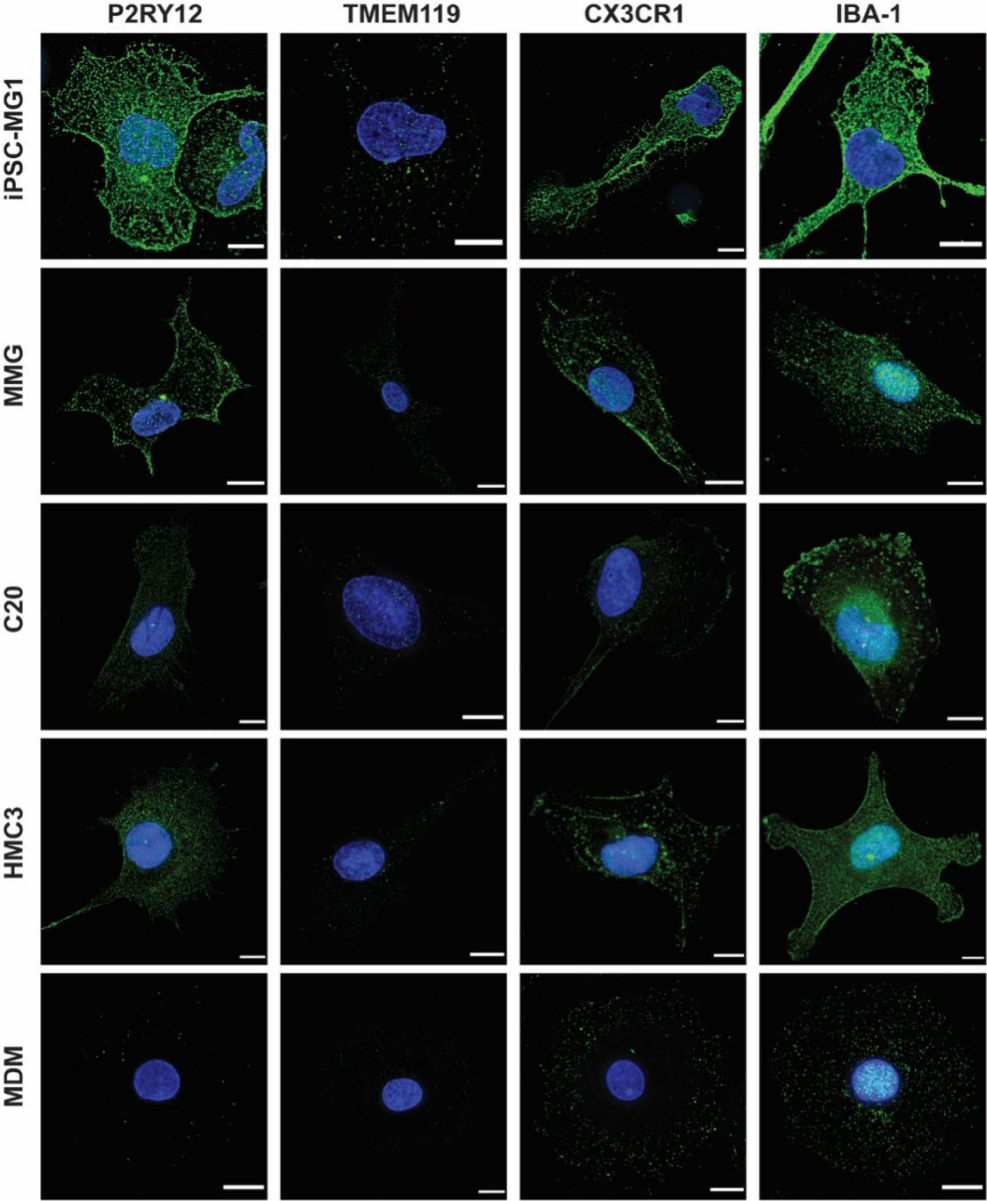
Immunofluorescence staining for microglial markers P2RY12, TMEM119, CX3CR1 and IBA-1 for the four microglial cell types and MDMs as control. Cells were fixed, permeabilized, and then stained with DAPI (blue) and antibody (green) for the different markers (P2RY12, TMEM119, CX3CR1 and IBA-1). Scale bar = 10 µm

We next sought to characterize each microglia model for expression of proteins typical of human microglia. We included monocyte-derived macrophages (MDM) as a comparator cell type. We first examined surface expression of traditional myeloid markers CD11b, CX3CR1, CD68 and CD45 by flow cytometry. iPSC-MG, MMG and MDM expressed each of these markers as expected, while C20 and HMC3 demonstrated very little or no expression of CD11b and CD45 (Fig. 2). Microglia in brain and spinal cord are characterized by high expression levels of the chemokine receptor CX3CR1, a feature that is sometimes utilized to visualize microglia in vivo [46]. Notably, CX3CR1 was highly expressed on iPSC-MG and less so on the other model cells (Fig. 2). This initial flow panel highlights the expression of typical myeloid markers by each cell type, with the exception of the poor expression of CD45 and CD11b by C20 and HMC3 cells. We next performed immunofluorescence staining for commonly used microglia markers CX3CR1, IBA-1, P2RY12, and TMEM119, (Fig. 3). All microglia model cells and MDM were positive for IBA-1 and CX3CR1 as is typical for microglia. However, expression of these markers was also seen at low levels for MDM, confirming that these are myeloid markers and are not limited to expression in microglia as previously established [47–49]. P2RY12 and TMEM119 are more specific markers that have been used to distinguish microglia from infiltrating macrophages in the CNS [47, 50]. We found that P2RY12 was present in each of the microglial model systems evaluated, although somewhat more prominent in iPSC-MG and MMG than in the transformed lines, and was absent in MDM. TMEM119 was similarly present in all four cell types, and very faintly stained MDM (Fig. 3). Thus, all four cell types represent authentic microglia as assessed by expression of these microglia-specific markers.

### Gene expression analysis of microglia model cells compared to human adult and fetal microglia

**Fig. 4:**
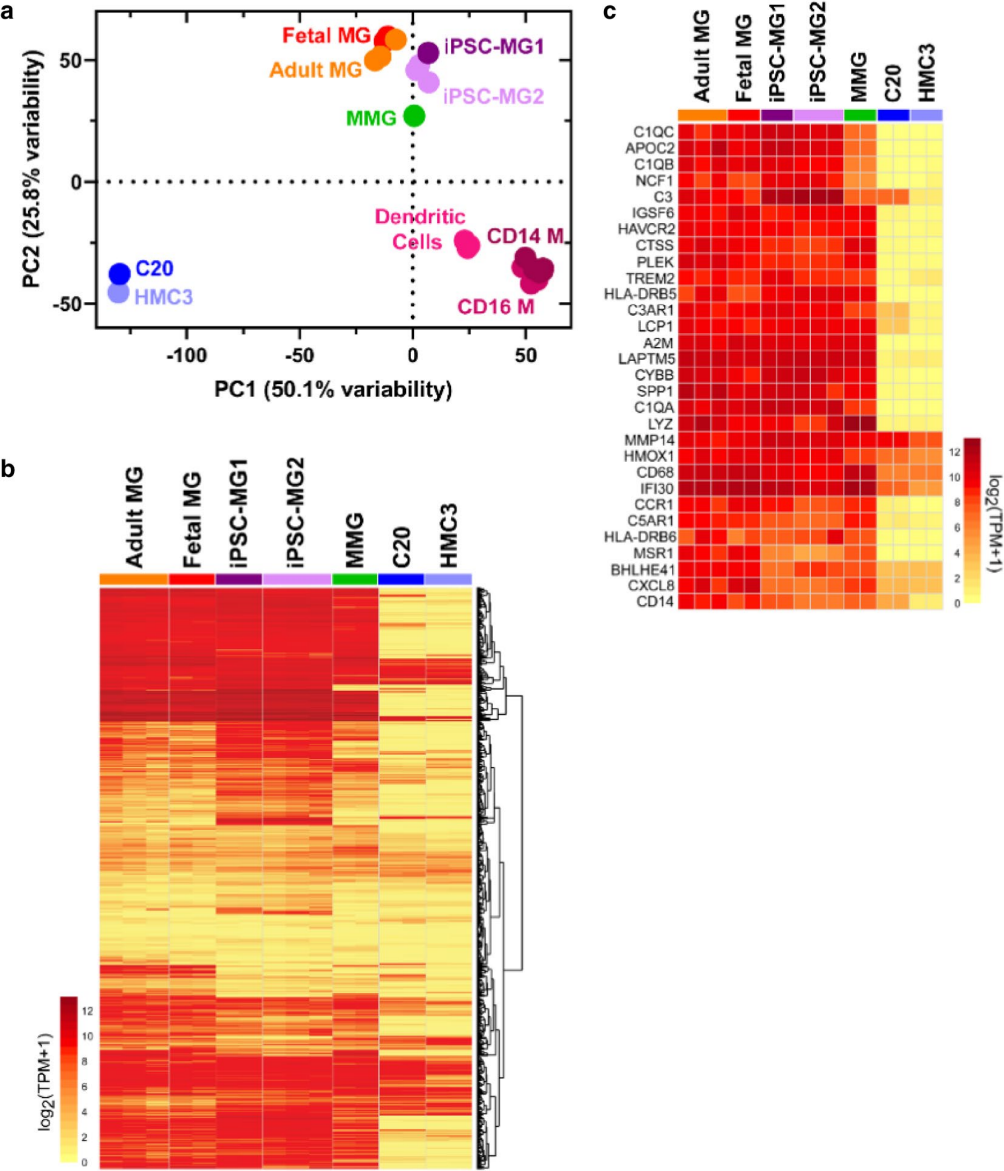
Gene expression analysis of iPSC-MG and MMG resemble Human Microglia. **a** PCA of iPSC-MG from this study (purple), iPSC-MG2 from the Blurton-Jones lab (magenta), MMG (green), human fetal microglia (red), human adult microglia (orange), dendritic cells (pink), CD14 monocytes (maroon), CD16 monocytes (rose), C20 (blue) and HMC3 (light blue) (TPM1 ≥ 1, n = 38,479). **b** Heatmap of 780 microglia-enriched genes as identified in other studies [45, 51]. **c** Heatmap signature of the 30 most highly expressed human primary adult microglia genes

To characterize the four model microglia in more detail, we next evaluated gene expression in each model cell type and compared the results to published profiles of adult and fetal microglia. Model profiles were also compared with CD16+/CD14− inflammatory monocytes (CD16M), CD14+/CD16− monocytes (CD14M) and myeloid dendritic cells in order to assess myeloid molecular character [43]. Furthermore, iPSC-MG transcriptomes generated in our laboratory were compared with iPSC-MG generated by the Blurton-Jones laboratory to assess and control for consistency in production methodologies [45]. We will refer to iPSC-MG results from the current study as iPSC-MG1 and those from the Blurton-Jones laboratory as iPSC-MG2 in relevant figures for clarity, while the term iPSC-MG applies to all preparations. Principle component analyses (PCA) revealed three distinct clusters consisting of 1) the transformed microglia cell lines (C20 and HMC3), 2) blood myeloid cells (CD16M, CD14M and dendritic cells), and 3) primary human microglia, iPSC-MG and MMG (Fig. 4a). Gene expression profiles from the Blurton-Jones lab (iPSC-MG2, dark purple, 3 independent preparations) [45] and from this study (iPSC-MG1, showing two independent preparations, light purple) were extremely similar by PC1 analysis (50.1% variability) and separated slightly on the PC2 axis (25.8% variability) (Fig. 4a), demonstrating a strong degree of similarity of iPSCs prepared in different laboratories. We noted the clustering of iPSC-MG (purple) and MMG (green) with adult (orange) and fetal (red) microglia, indicating that both of these model cells share significant gene expression characteristics with authentic microglia, with some separation of MMG along the PC2 axis. In contrast, C20 and HMC3 cell lines were widely dissimilar from adult and fetal microglia, as demonstrated by their separation along both PC1 and PC2 axes. In order to further analyze the relation of microglia model systems to primary human microglia, we restricted the comparison to 780 microglia-enriched genes compiled from the analysis of primary microglia gathered from brain tissue resection of 19 individuals (Fig. 4b and Additional file 2: Table S1) [51]. The hierarchically clustered heat map of gene expression in Fig. 4b provides further evidence of the similarity of C20 and HMC3 cell lines and of the distinct gene signatures shared by iPSC-MG and MMG with adult and fetal microglia. This analysis also reinforces the similarity of iPSC-MG profiles previously published with the iPSC-MG in the present study (iPSC-MG2 in Fig. 4b). Next, we restricted the analysis to the 30 most highly expressed genes in the primary microglia data (Fig. 4c) [43]. This analysis again illustrates a strikingly similar relationship between gene expression in iPSC-MG and gene expression in primary human adult and fetal microglia, whereas the human transformed microglial cell lines demonstrated stark differences (Fig. 4c). The relationship of specimens was quantified using a correlation matrix of TPMs (transcripts per million) from the subset of 780 microglia-enriched genes (Additional file 3: Fig. S2). When compared with primary human adult microglia, C20 and HMC3 generated Pearson’s coefficients demonstrating very weak correlation, avg 0.13 ± 0.02 and 0.05 ± 0.01 respectively. In contrast, significant conservation of microglia signature expression between primary adult human microglia and iPSC-MG (0.63 ± 0.12) and MMG (0.75 ± 0.11) was confirmed. iPSC-MG (taken together) were more similar to adult microglia than HMC3 and C20 (p-value = 1.21 × 10^−5^, Wilcoxon rank-sum test on the correlation coefficients in Additional file 3: Fig. S2 for iPSC-MG vs. AMG and HMC3 plus HMC3 vs. AMG). MMG were also more similar to adult microglia than HMC3 and C20 (p-value = 8.11 × 10^−5^, Wilcoxon rank-sum test on the correlation coefficients in Additional file 3: Fig. S2 for MMG vs. AMG and HMC3 plus HMC3 vs. AMG).

**Fig. 5:**
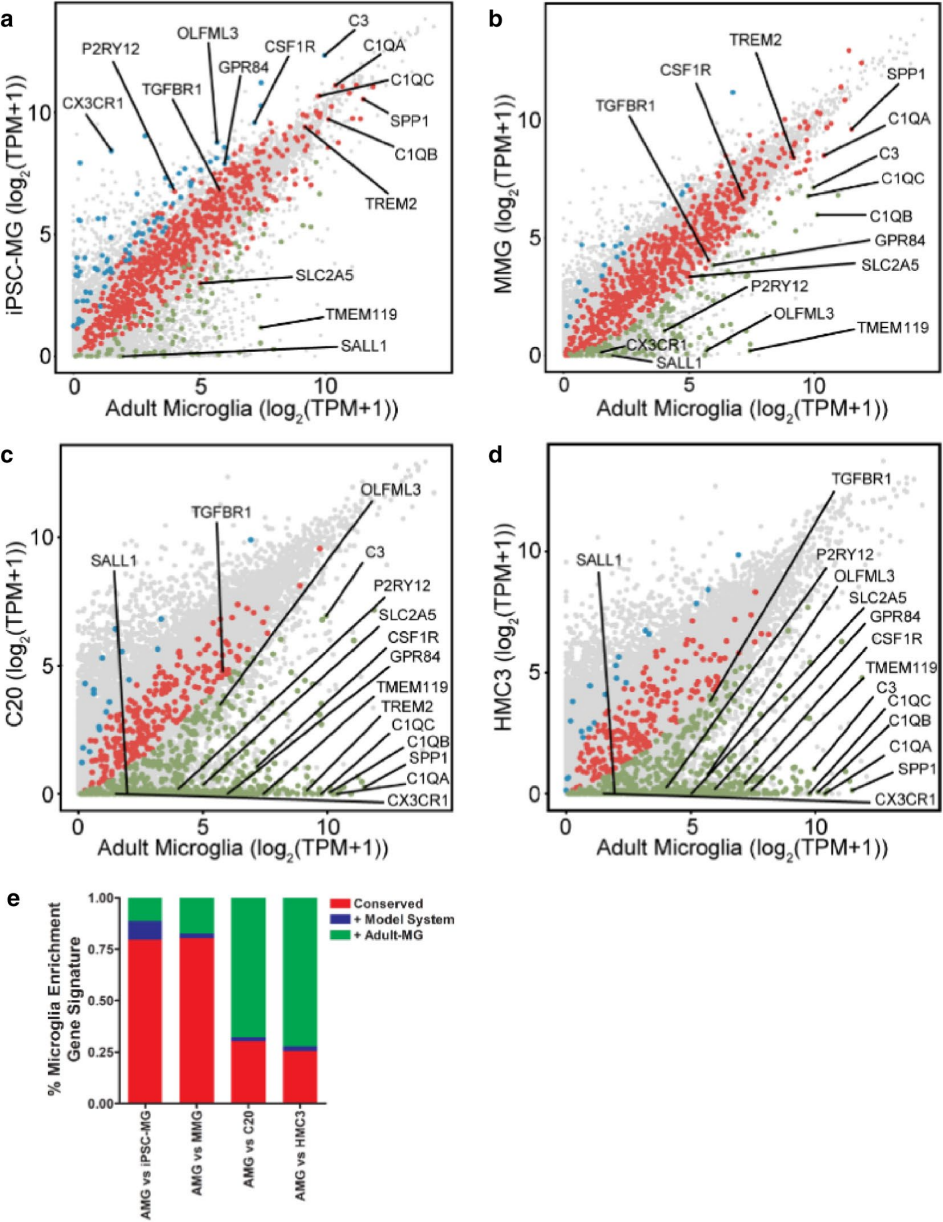
Scatter plots comparing mRNA expression levels from microglia model systems. Signature comprised of 780 microglia-enriched genes are displayed as colored dots. Conserved gene expression is shown in red; genes more highly expressed in human adult microglia are shown in green, and genes more highly expressed in model systems are shown in blue (FC > 2, FDR ≤ 0.05). A panel of 15 core primary human adult microglia genes are labeled with text. **a** iPSC-MG vs Adult-MG, **b** MMG vs Adult-MG, **c** C20 vs Adult-MG, **d** HMC3 vs Adult-MG. **e** Stacked bar plot showing conservation and divergence of 780 primary human adult microglia enriched transcripts from putative microglia model systems. Red corresponds to conserved signatures with adult microglia (AMG), green are expressed at higher levels in AMG, blue at higher levels in the indicated model cell type

Results above demonstrate that both MMG and iPSC-MG are more similar by gene expression profiling to primary microglia than the transformed cell models C20 and HMC3. We next sought to analyze differences in selected characteristic microglial genes when MMG and iPSC-MG were individually examined in a scatter plot for similarity to adult microglia (Fig. 5). Using the subset of 780 microglia-enriched genes, this figure depicts similarity of expression with adult microglia as red circles, while those genes that are more highly expressed in the indicated model line are shown in blue, and those that are more highly expressed in adult microglia are shown in green. We chose an additional fifteen core human microglia signature genes (including *Tmem119* and *P2ry12*) and indicate their positions in the similarity plot with black lettering [37, 45, 47, 51, 52]. The identity and reason for selection of these fifteen genes is outlined in Additional file 4: Table S2. Significant similarity can be appreciated between iPSC-MG and adult microglia in Fig. 5a, and between MMG and adult microglia in Fig. 5b, as represented by the red circles along the diagonal. In contrast, many typical microglial genes were more highly expressed in adult microglia than in C20 (Fig. 5c) or HMC3 (Fig. 5d), as represented by the green circles along the x-axis. We note also that the fifteen genes listed in Additional file 2: Table S2 are almost entirely dissimilar in expression for C20 and HMC3 as compared to adult microglia. The percent of conserved expression between model cells and adult microglia is shown in another way in Fig. 5E, demonstrating that > 75% of the panel of 780 microglial genes were expressed at similar levels in MMG and iPSC-MG, while the transformed lines showed major differences in microglial gene expression.

### Expression of genes relevant to HIV replication and restriction

**Fig. 6:**
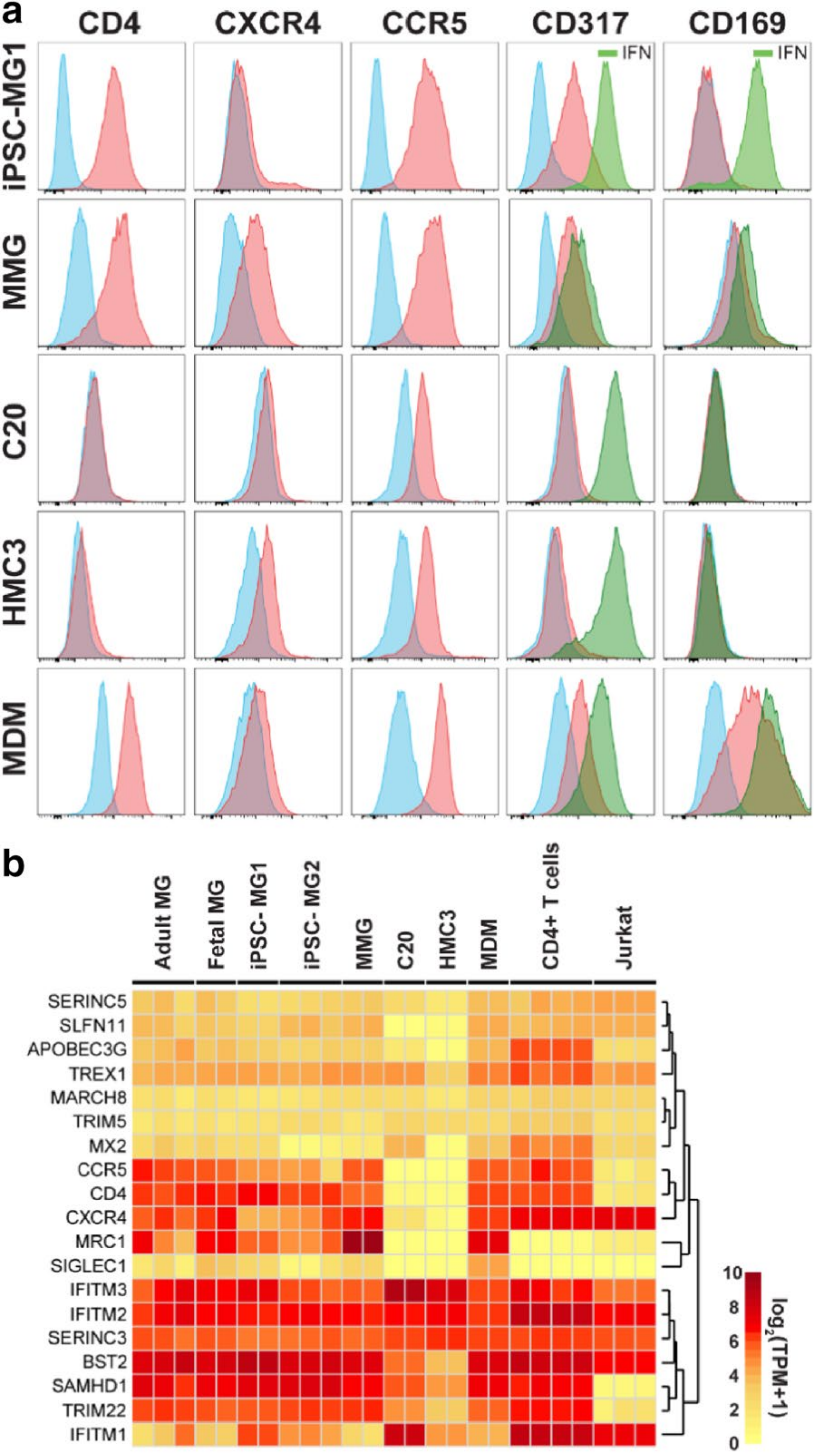
Human microglia model expression profile of selected factors related to HIV-1 biology. **a** Histograms displaying cell surface expression of CD4, CXCR4, CCR5, CD317, and CD169 for the four microglial model cells and MDM. Isotype controls are shown in blue, specific staining at baseline in red. IFN stimulation was pursued for CD317 and CD169, with post-stimulation histograms depicted in green. **b** Heatmap of HIV-1 receptor, coreceptor and restriction factor gene expression, with color scale of expression levels indicated

A major goal of this study was to identify microglia model systems that are most appropriate for studies of HIV infection, transmission, and pathogenesis. In particular, expression of CD4 receptor, CCR5 coreceptor, and restriction factors that limit HIV infection or spread are almost certainly going to affect results when microglia are modeled in vitro. We noted that CD4 was absent from C20 and HMC3 cells (Fig. 6a), consistent with previous reports [29, 32]. iPSC-MG and MMG on the other hand, expressed significant levels of CCR5 and CD4. We examined Siglec-1 (CD 169) and tetherin (CD317) expression, two interferon (IFN)-inducible proteins relevant to HIV restriction (tetherin) or particle uptake and transmission (Siglec-1) [53, 54]. iPSC-MG, MMG and MDM expressed CD317 and CD169 in the absence of IFN-stimulation, while levels were enhanced following treatment with Type 1 IFN. In contrast, HMC3 and C20 cell expressed no CD317 or CD169 in the unstimulated state (Fig. 6a). IFN stimulation led to expression of CD317 but not CD169 in these transformed cell lines. Thus, this initial antigen panel identified differences in expression of CD4, CD317, and CD169 by C20 and HMC3 cells as compared with iPSC-MG and MMG. In order to more completely evaluate HIV-relevant gene expression we quantified mRNA levels for 19 selected genes (Fig. 6b). The absence of CD4 and Siglec-1 and lower levels of BST-2/tetherin seen at the protein level for C20 and HMC3 was confirmed at the RNA level. Expression levels of other HIV-1 restriction factors including *TRIM5*, *APOBEC3G*, and *SAMHD1* seen in iPSC-MG and MMG were generally similar to those of adult and fetal microglia. Lower levels of transcripts for *SAMHD1*, *BST2*, and *APOBEC3G* were detected in C20 and HMC3 cells as compared with adult microglia and iPSC-MG. We point out that CCR5 surface expression in C20 and HMC3 was low but present by flow cytometry as compared with other characterized model systems (Fig. 5a), and yet RNAseq analysis repeatedly generated zero reads for *CCR5* (Fig. 6b). While this was not expected, it was perhaps due to its expression hierarchy in these transformed lines. Taken together, analysis of HIV-relevant gene expression indicate that iPSC-MG and MMG express this subset of genes at similar levels when compared with CNS microglia, and that significant differences in expression of receptor and some restriction factors are found in C20 and HMC3 cells. From the specimens analyzed, iPSC-MG were most similar in regards to HIV-1 related factor expression to adult microglia (p-value = 1.15 × 10^−7^, Wilcoxon rank-sum test on paired correlations of restriction factor expression in Fig. 5b for iPSC-MG vs. AMG and HMC3 plus C20 vs. AMG). In contrast, while MMG express many of these factors, the comparative profile with adult microglia (MMG vs AMG and HMC3 plus C20 vs AMG) does not reach statistical significance (p-value = 0.8916). The similarities in expression of HIV-relevant genes further support the use of iPSC-MG and MMG in studies of HIV replication and pathogenesis.

To confirm key results seen with gene expression analysis, we performed quantitative RT-PCR. Results for *P2ry12*, *Tmem119*, *Aif1*, *Cx3cr1*, *Cd4*, *Ccr5*, *Siglec1*, and *Bst2* are shown in Additional file 5: Fig. S3. These results confirmed the major differences outlined above, including expression of typical microglial marker message in iPSC-MG and MMG, and lack of expression of *Cd4*, *P2ry12*, and *Tmem119* in C20 and HMC3.

### Susceptibility to HIV-1 infection

**Fig. 7:**
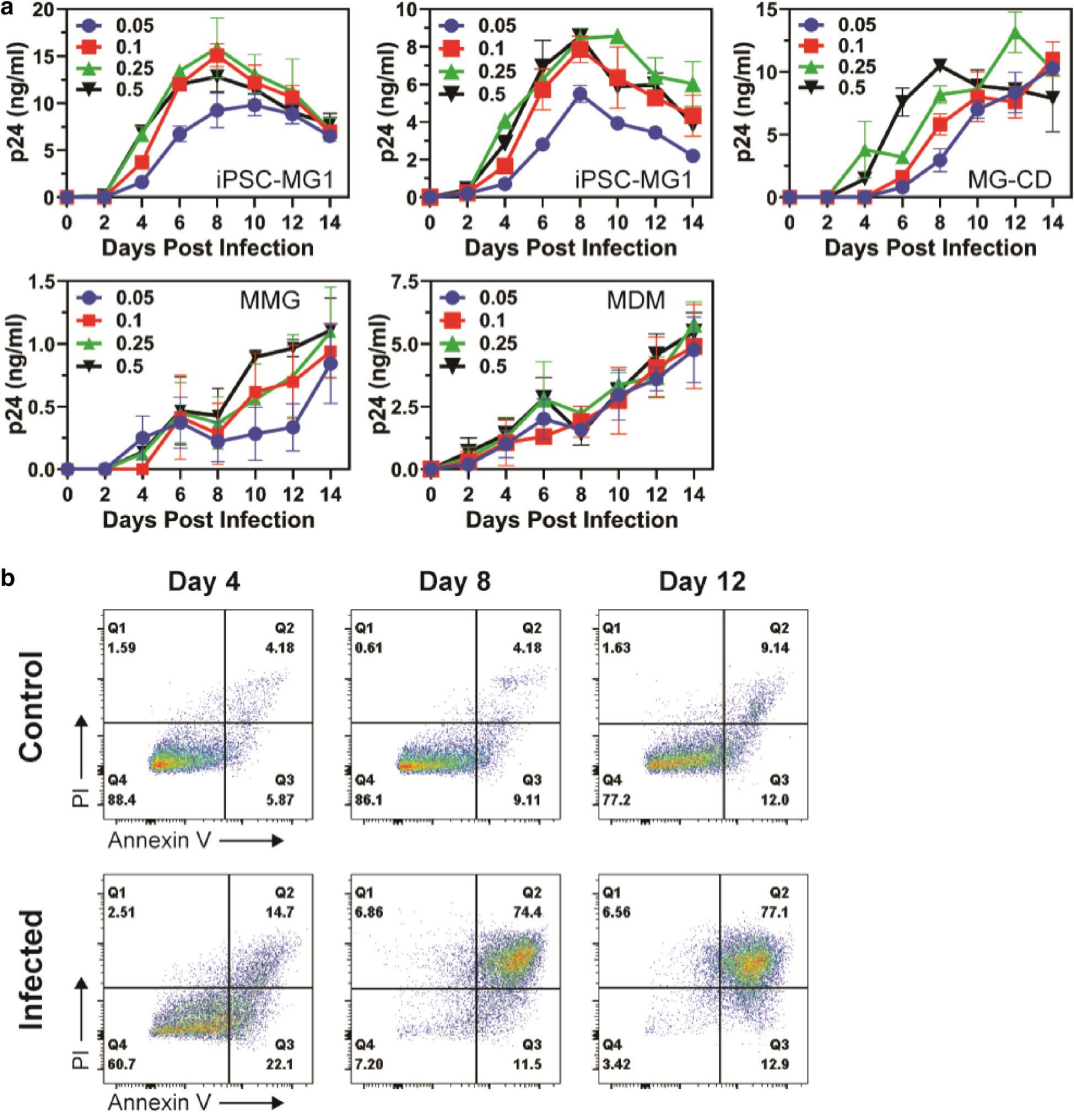
HIV replication in microglia model systems. **a** iPSC-MG1 (two panels), MG-CD, MMG and MDMs were infected with HIV-1_BaL_ at the indicated MOIs. Culture supernatants from HIV infected iPSC-MG1, MG-CD, MMG and MDMs were collected at days 0, 2, 4, 6, 8, 10, 12 and 14 post-infection and measured by p24 antigen capture ELISA. MG-CD are iPSC-MG obtained from Cellular Dynamics. **b** iPSC-MG1 were infected with HIV-1_BaL_ at an MOI of 0.25 and assayed by flow cytometry for apoptosis/necrosis at days 4, 8 and 12 post-infection by annexin V and propidium iodide co-staining

We next performed a head-to-head comparison of HIV-1 infection using these four model sources of microglia. Microglia were infected with HIV-1_BaL_ at a range of multiplicity of infection (MOI) from 0.05 to 0.5, and the release of virus in the form of p24 antigen was measured over time. iPSC-MG were susceptible to productive infection with HIV‐1_BaL_ without the need for pseudotyping (Fig. 7a, both iPSC-MG1 panels). iPSC-MG infection produced virus that peaked at day 8 and then declined. MMG, in contrast, continued to produce virus over the two week experiment, and produced lower levels of virus (Fig. 7a, MMG panel). This pattern was very similar to that seen with MDM infected with HIV-1_BaL_ (Fig. 7a, MDM panel). Both patterns were consistent at a range of MOI, although the magnitude of particle release was diminished at the lowest MOI for iPSC-MG and MMG. After seeing the difference in growth curves between iPSC-MG and MMG, we obtained iPSC-MG from a commercial source (Cellular Dynamics), and repeated the experiment. These iPSC-MG, for this figure termed MG-CD, presented a pattern that seemed to bridge the MMG and iPSC-MG findings: at lower MOI, the ongoing release of virus was consistent with that of MMG, while at the highest (0.5) MOI the pattern closely resembled that of iPSC-MG (Fig. 7a, MG-CD panel, note inverted black triangles for highest MOI). To further illustrate this point, we overlayed results from a lower MOI curve from our iPSC-MG with that of the commercial MG-CD culture at high MOI, and they were remarkably similar (Additional file 7: Fig. S5). This suggested to us that the differences seen in the growth curves likely relate to the efficiency of initial infection, i.e. our iPSC-MG were more readily infected than the commercial cells, and upon infection at a high MOI both sources of iPSC-MG showed a peak followed by a decline.

We noted enhanced cytotoxicity by visual inspection for iPSC-MG at the time of peak release of virus (day 8), a characteristic not observed for MMG or for MDM, and less prominent in the commercial MG-CD (data not shown). Because this could explain the dropoff in p24 release seen in infected cultures, we looked for markers of apoptosis and cell death using annexin V and propidium iodide staining in HIV-infected iPSC-MG over time. Control cells showed modest degrees of apoptosis and cell death over time (Fig. 7b, control row). HIV-infected iPSC-MG, in contrast, showed enhanced early apoptosis at day 4 as indicated by higher levels of annexin V staining, and by day 8 had progressed to significant levels of late apoptosis (74% of cells positive for both annexin V and propidium iodide). This pattern persisted at day 12. We conclude that apoptotic cell death is the likely explanation for the decline in p24 in HIV-infected iPSC-MG cultures after day 8.

C20 and HMC3 lack CD4 and were not able to be infected by HIV‐1_BaL_ as expected (Additional file 6: Fig. S4A). These transformed microglial lines were infectable with VSV-pseudotyped HIV-1, however, and thereafter demonstrated sustained p24 release (Additional file 6: Fig. S4B). Of note, C20 and HMC3 divided much more rapidly than iPSC-MG, MMG, or MDM, requiring splitting of infected cell cultures over the course of these experiments, while maintenance of cultures over this time period for iPSC-MG and MMG was feasible without splitting cells.

**Fig. 8:**
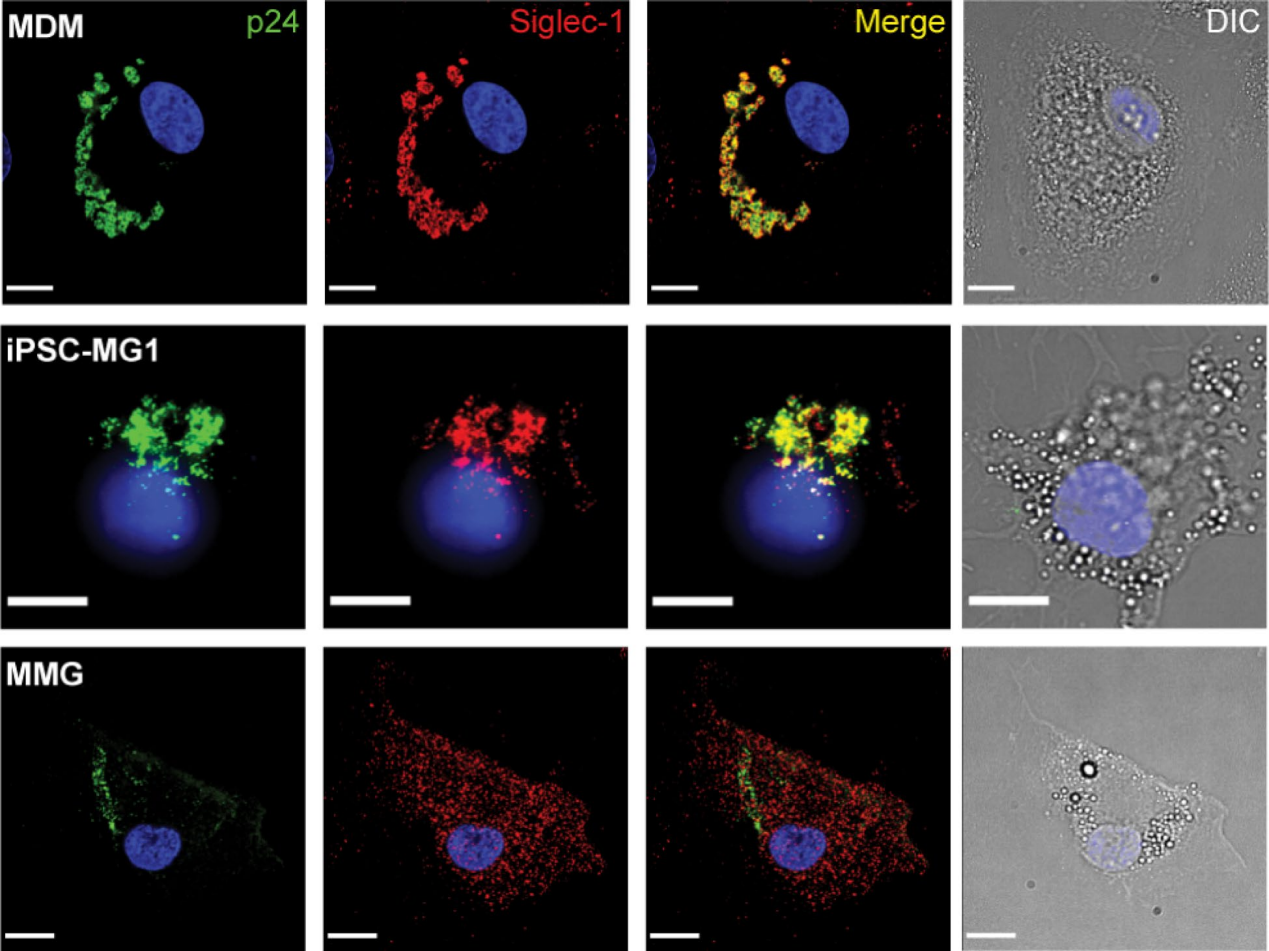
HIV particle capture and Siglec-1 colocalization with p24 in HIV-1-infected MDM (top row), iPSC-MG1 (middle row), and MMG (bottom row). Immunofluorescence microscopy is shown examining Siglec-1 colocalization with p24. The cell types were infected with a biological stock of HIV-1_BaL_ at an MOI of 0.5. On day 8 post infection, cells were fixed and immunolabeled for HIV-1 p24 (green), Siglec-1 (red) and DAPI was used as a nuclear marker. Scale bar = 10 μm

MDMs infected with HIV-1 typically demonstrate a prominent intracellular compartment termed the virus-containing compartment (VCC) [54–58]. We have shown that the formation of this compartment depends upon Siglec-1, and that Siglec-1 is a prominent component of the VCC [53]. We next examined iPSC-MG and MMG following infection with HIV-1 BaL to determine if the characteristic VCC of MDMs is also found in microglia. At day 10 post-infection with HIV-1, prominent VCC were identified in MDM as indicated by intracellular collections of p24 (green) colocalizing with Siglec-1 (red) (Fig. 8, top panels). iPSC-MG similarly demonstrated significant co-localization of p24 with Siglec-1 (Fig. 8, middle panels). However, the distribution of p24 and Siglec-1 was more peripheral, and the compartment morphology differed from that of MDMs, suggesting a common mechanism of particle capture by Siglec-1 but potential differences in internalization and VCC formation in iPSC-MG. MMG examined in this manner failed to show any consistent concentration and colocalization of Siglec-1 with HIV-1 virions. HIV-1-infected MMG instead revealed a diffuse localization of Siglec-1 and a peripherally located virus without any evident VCC (Fig. 8, bottom panels). We conclude that while iPSC-MG and MMG are both robust microglia model cells for investigation of HIV-microglia interactions, there are important differences in viral replication characteristics and particle capture/internalization that will require further investigation.

## Discussion

Efforts to understand HIV-1 transmission and pathogenesis in the CNS will require a detailed understanding of infection of resident CNS microglia, which are thought to be the main cell type infected by HIV in the human brain [59, 60]. Transformed microglial cell lines represent one option as model systems for studying HIV interactions. The HMC3 line is a transformed microglial line that has been widely used and is the only human microglia cell line available from ATCC [28, 29]. C20 cells and other transformed primary human microglial lines have been utilized as models for studying HIV kinetics and latency in human microglial cells [61, 62]. MMG and iPSC-MG have been shown to recreate authentic microglia phenotypically and functionally, and offer potential advantages for studies of HIV-microglia interactions [33, 36, 43]. In approaching how to best study the interaction of HIV-1 with microglia, we reasoned that a comprehensive comparison of these four microglial models examining microglial antigen expression and gene expression together with antigens and genes of interest to HIV replication would be useful. Furthermore, we sought to perform an initial look at HIV replication in each model cell type.

We did not differentiate the models significantly by morphology. Each of the cell types examined were adherent, had ovoid central bodies surrounding the nucleus and cellular extensions (ramifications) typical of microglia. An important difference in growth characteristics was immediately apparent, however, in that HMC3 and C20 cells proliferated rapidly, requiring splitting of plated cells every 72 h, while iPSC-MG and MMG were terminally differentiated with a very low incidence of dividing cells and could be maintained in culture for 2–3 weeks or more. In this characteristic iPSC-MG and MMG more closely resemble the division of microglia in the adult brain, where at any given time only a small minority (< 2%) are actively dividing, and doing so with a cell-cycle length of 32 h [63].

Antigenic profiling revealed that each of the four cell types expressed characteristic myeloid markers, with the exception of low-to-absent expression of CD11b and CD45 on the two transformed cell lines, and high expression of CX3CR1 in iPSC-MG. Each cell type also expressed markers typically used to identify microglia, including P2RY12 and TMEM119. Therefore, little differentiation between the models can be made on the basis of antigenic profiling using these selected microglial markers alone. Gene expression profiling, however, revealed striking differences. HMC3 and C20 cluster together by PCA (Fig. 4a) and are similar by multiple additional analysis, and are far removed in gene expression patterns from adult (or fetal) microglia. We conclude from gene expression profiling that both iPSC-MG and MMG appear to be more representative of authentic microglia than the two transformed microglial lines, and on this basis would both be reasonable models for further use.

Central to this study was the examination of expression of genes in microglia that are required for or inhibit HIV replication. In this analysis, it became even more apparent that iPSC-MG and MMG offer a more authentic representation of expression patterns seen in CNS microglia. C20 and HMC3 cells lack HIV receptor CD4, thus requiring pseudotyping for experiments examining any aspect of HIV infection. Baseline levels of tetherin were not detected at the protein level in C20 or HMC3, although tetherin could be induced by IFN stimulation. Strikingly, the cell surface lectin Siglec-1 was absent in these cells even following IFN stimulation, whereas iPSC-MG1 and MMG demonstrated IFN-stimulated expression of Siglec-1. Given the growing recognition that Siglec-1 can capture HIV-1 particles and enhance transmission to susceptible target cells [53, 64–66], the role of Siglec-1 in particle capture and transmission by microglia is an important area to study and will require appropriate model cells expressing this lectin. However, expression levels of Siglec-1 at baseline (in the absence of IFN-stimulation) were low even in iPSC-MG1 and MMG. We note that expression of other *Siglecs* at the mRNA level were much higher in iPSC-MG at baseline, including *Siglec* 5, 10, 14, and 16. There is currently no data on the role of these highly-expressed Siglecs in HIV-1 interactions, it will be interesting to dissect this in future studies. HIV-1 restriction factor expression levels in adult microglia were more similar to that of iPSC-MG and MMG than for C20 and HMC3. This was most striking for *APOBEC3G*, *BST2*, and *SAMHD1*, with diminished restriction factor expression in C20 and HMC3. In summary of the examination of HIV-related genes, expression of receptor *CD4*, *BST2*, *SIGLEC1*, *APOBEC3G*, and *SAMHD1* by the transformed microglial lines differed markedly from adult microglia, favoring the iPSC-MG and MMG models as more appropriate models for studies of HIV-microglia interactions.

Infection of model microglial cells with HIV-1_BaL_ revealed some differences in particle production and release. iPSC-MG produced high levels of virus release that peaked on day 8 and then waned over the following 6 days, while MMG continuously released virus at lower levels over the two-week period examined. In this manner, MMG were more similar to the pattern typically seen with MDM. It has been demonstrated previously that while replication of many HIV isolates in human brain tissue-derived microglia closely resembles replication in macrophages, not all isolates follow this pattern [67]. The reason for the decline of virus release in iPSC-MG1 culture over time was shown in our study to be from induction of apoptosis and resulting cell death. We note that primary human microglia derived from human brain infected with the same HIV-1_BaL_ viral isolate exhibited a pattern of peak virus released followed by a plateau and then decline, a curve that also resembles what we observed [68]. A more recent report of infected iPSC-MG employing a different viral strain also demonstrated a sharp rise in p24 release followed by a plateau, but did not extend the growth curve beyond day 15 of the plateau to assess potential decline [69].

iPSC-MG are increasingly being used to model microglia and in multicellular systems that attempt to recreate brain architecture [43], and are likely to be utilized increasingly in the study of HIV interactions. To further evaluate the HIV replication pattern we observed in iPSC-MG, we obtained iPSC-MG from a commercial source and repeated infections with HIV-1_BaL_. Although these iPSC-MG exhibited a lower and more sustained release of p24 than our iPSC-MG cultures developed in-house when infected at low MOI, the growth curve at the highest MOI in these commercial cells was nearly identical to those we had observed at lower MOI in our cells (Fig. 7a and Additional file 7: Fig. S5), suggesting to us that differences observed were likely due to a higher initial efficiency of microglial infection of our in-house produced cells. It will be interesting in future studies to examine gene expression in iPSC-MG and MMG over the course of HIV infection to determine if there are differences in cellular pathways that lead to cellular activation or to apoptosis versus cell survival over time, or if the key determinant is related primarily to the efficiency or magnitude of initial infection.

Another striking difference between iPSC-MG and MMG was the marked colocalization between p24 and Siglec-1 seen in iPSC-MG, which was not seen in MMG. This cannot be explained by initial Siglec-1 expression levels, as these were similar between the two in the unstimulated state. However, we note that IFN stimulation resulted in marked upregulation of Siglec-1 in iPSC-MG, while only a small upregulation was seen in MMG (Fig. 6). iPSC-MG Siglec-1/p24 colocalization itself differed from that of MDM, with a more peripheral distribution and less colocalization of p24 and Siglec-1 in deep compartments consistent with the VCC. These differences in particle release and VCC formation will also warrant further investigation. As noted above, other Siglecs are expressed at high levels in microglia and are potential candidates for HIV-1 particle capture, while Siglec-1 levels on microglia are low but are substantially enhanced by IFN stimulation.

Studies here suggest that two microglial models, iPSC-MG and MMG, are most suitable for studying HIV-microglia interactions in vitro, with iPSC-MG showing somewhat greater similarity to adult microglia. Much work remains to be done in understanding HIV interactions with microglia, including defining changes in microglial gene expression following infection, understanding the functional role of restriction factors in microglia, establishing factors that promote transmission of HIV-1 from infected microglia to uninfected microglia or infiltrating macrophages and T cells, and the development of latency in microglia. The ultimate aim for studies of in vitro models of HIV-microglia interactions is to elucidate the origins of HIV-induced CNS disease and provide insights leading to treatments or preventive measures for HAND. Microglia are central to this mission, and therefore tractable sources of human microglia are essential. Cerebral organoids have been developed from iPSCs and can recreate three-dimensional cellular relationships in the CNS [70, 71]. We can anticipate that some of the findings from studies of HIV-microglia interactions performed in isolation will translate to the organoid setting, while others will likely differ in the context of interactions with multiple cell types and with extracellular matrix components. As HIV studies move into these more complex 3D models, the use of sources of authentic microglia will be essential.

## Conclusions

iPSC-MG and MMG are very similar to authentic microglia in overall gene expression and in expression of HIV-related genes, and are therefore better suited for HIV-related studies than immortalized microglial cell lines. Studies of HIV interactions with microglia will benefit from the use of these model systems. Despite overall similarities in gene expression, HIV replication differed between iPSC-MG and MMG, and the basis for this difference requires further study.

## Methods

### Induced pluripotent stem cells (iPSCs)

The iPSC72.3 line was derived from primary human foreskin fibroblasts (HFFs) cultured from neonatal human foreskin tissue. Tissues were obtained through the Department of Dermatology, University of Cincinnati. iPSC72.3 was generated by the CCHMC Pluripotent Stem Cell Facility, approved by the CCHMC institutional review board and previously characterized [72, 73]. iPSC72.3 had a normal male karyotype and differentiated into endoderm, mesoderm, and ectoderm lineages in an in vivo teratoma assay. Induced pluripotent stem cells were grown in feeder-free conditions in six-well Nunclon surface plates (Nunc) coated with Matrigel (BD Biosciences) and maintained in TeSR-E8 media (Stem Cell Technologies) at 37 °C with 5% CO_2_. Cells were checked daily for differentiation and were passaged every 4 days using Gentle Cell Dissociation Reagent (Stem Cell Technologies). iSPC72.3 were checked for karyotype and routinely checked for mycoplasma.

### Isolation and maturation of monocyte-derived macrophages (MDMs)

Human peripheral blood mononuclear cells (PMBCs) were isolated from fresh heparinized blood by Ficoll-Hypaque gradient centrifugation. Buffy coats were pooled, and platelets removed by washing repeatedly with phosphate-buffered saline (PBS). Monocyte enrichment was performed by indirect magnetic labeling using the Pan Monocyte Isolation Kit (Miltenyi Biotec) according to manufacturer’s protocol. Enriched monocytes were plated on poly-d-lysine coated plates (Corning) and type 1 rat tail collagen coated 35 mm MatTek dishes (MatTek). Monocytes were maintained in RPMI-1640 supplemented with 10% FBS (Lot No. F-14070, Atlanta Biologicals), 100 µg/mL streptomycin, 100 U/mL penicillin, 2 mM GlutaMAX, and 5 ng/mL GM-CSF (Peprotech). Monocyte cultures were maintained in GM-CSF supplemented media for 7 days to mature cells to MDMs. Media was replaced every 3–4 days.

### Isolation and maturation of monocyte-derived microglia (MMG) from whole human blood

Peripheral blood mononuclear cells (PBMCs) were isolated from whole blood of HIV seronegative donors, and monocytes prepared as described above. MMG were generated from isolated monocytes using methods modified from those previously described [34, 37]. To induce the differentiation of MMG, monocytes were cultured in RPMI-1640 supplemented with 1X GlutaMAX (Life Technologies), 1% penicillin/streptomycin and a mixture of the following human recombinant cytokines: M-CSF (10 ng/mL; Peprotech), GM-CSF (10 ng/mL; Peprotech), NGF-β (10 ng/mL; Peprotech), CCL2 (100 ng/mL; Peprotech), and IL-34 (100 ng/mL; Peprotech) under standard humidified culture conditions (37 °C, 5% CO_2_). The monocytes were incubated with 1% serum for the first 3 days, and thereafter, every third day, cells were supplemented with fresh serum-free media containing M-CSF, GM-CSF, NGF-β, IL-34 and CCL2. Cells were cultured for 14 days. Characterization experiments were performed on day 14.

### Differentiation of iPSCs to hematopoietic progenitor cells (HPCs)

Differentiation of iPSCs to HPCs was performed using the STEMdiff© Hematopoietic Kit (Stem Cell Technologies, 05310). iPSCs were cultured in TeSR-E8 supplemented with 0.5 µM Thiazovivin on 1 mg/mL hESC-qualified Matrigel-coated (Corning, 354227) 6-well plates. On the day prior to differentiation, iPSCs were passaged with Gentle Cell Dissociation Reagent (Stem Cell Technologies, 07174) and aggregates of approximately 100 cells were plated onto several 10 cm^2^ dishes at a target density range of 5–10 aggregates per cm^2^. On the following day, plates containing between 80 and 100 total colonies (approximately 2 per cm^2^) were chosen to proceed with differentiation protocol. On day 0, TeSR-E8 media supplemented with 0.5 µM Thiazovivin was replaced with 8 mL of basal media A containing a 1:200 dilution of supplement A. On day 2, a half culture basal media A change was performed. On day 3, basal media A was completely removed and replaced with basal media B containing a 1:200 dilution of supplement B. Half media exchanges were performed with basal media B on days 5, 7, 9, 10, 12 and 14. On days 12, 14 and 16 non-adherent cells were collected and centrifuged for 5 min at 300×*g*. Clarified supernatants were half media exchanged with basal media B on days 12 and 14 after harvesting HPCs. Pelleted cells were cryopreserved at a density of 1.5–2 × 10^6^ HPCs per mL of Bambanker (Wako, CS-02-001). Frozen HPCs were thawed rapidly by immersion in a 37 °C water bath and cultured immediately in cytokine supplemented microglia differentiation media and plated onto 1 mg/mL growth factor reduced (GFR) Matrigel-coated (Corning, 356230) 6-well dishes at 1 × 10^5^ cells per cm^2^.

### Maturation of HPC to iPSC- microglia (iPSC-MG)

HPCs are cultured at a density of 1 × 10^4^ per cm^2^ onto 1 mg/mL GFR Matrigel-coated 6-well plates in 2 mL of iPSC-MG media per well (DMEM/F12, 2% B27, 0.5% N2, 2% insulin-transferrin-selenium, 1X MEM Non-Essential Amino Acids Solution, 1X GlutaMAX, 400 µM 1-thioglycerol and 5 µg/mL human insulin). Prior to use, iPSC-MG media was supplemented with 100 ng/mL human IL-34, 50 ng/mL TGF-β1 and 25 ng/mL M-CSF (Peprotech). On days 2, 4 and 6 media were supplemented with the addition of 1 mL per well of iPSC-MG media with cytokines. On day 8, media was removed leaving behind 1 mL per well of conditioned media. Cells were centrifuged for 5 min at 300×*g*, media aspirated, and cells resuspended in 1 mL of iPSC-MG media with cytokines prior to addition back to wells. On days 10, 12 and 14 media were again supplemented with addition of 1 mL per well of iPSC-MG media with cytokines. On day 16, media was removed leaving behind 1 mL per well of conditioned media. Cells were centrifuged for 5 min at 300×*g*, media aspirated, and cells resuspended in 1 mL of iPSC-MG media with cytokines prior to addition back to wells. On days 18, 20 and 22 media were again supplemented with addition of 1 mL per well of iPSC-MG media with cytokines. On day 24, cells were resuspended in iPSC-MG media supplemented with a five-cytokine cocktail consisting of 100 ng/mL human IL-34, 50 ng/mL TGF-β1, 25 ng/mL M-CSF, 100 ng/mL CD200 and 100 ng/mL CX3CL1 in order to facilitate final maturation into iPSC-MG. On day 26 and 28, cells were fed by the addition of 1 mL per well of iPSC-MG media supplemented with five cytokine cocktail. By day 28 iPSC-MG were considered mature and used for further characterization and RNAseq analyses. Cells were maintained for a maximum of 2 weeks following the 8-day cycle of media addition and conditioned media maintenance described above.

Commercially available terminally differentiated iPSC-derived microglia were purchased from Cellular Dynamics (Fujifilm), termed MG-CD in this report. MG-CD were thawed according to the manufacturer’s instructions, and cultured in iPSC-MG media supplemented with 100 ng/mL human IL-34, 50 ng/mL TGF-β1 and 25 ng/mL M-CSF. MG-CD were infected with primary HIV-1 BaL together with iPSC-MG1, MMG and MDM as described.

### C20 and HMC3 cultures

The immortalized human fetal brain-derived microglia cell line HMC3 was obtained from the American Type Culture Collection (ATCC, CRL-3304). HMC3 were maintained in Eagle’s Minimum Essential Medium (EMEM) supplemented with 10% heat-inactivated fetal bovine serum (FBS). The immortalized human adult-derived microglia cell line C20 was a kind gift from David Alvarez-Carbonell (Case Western Reserve University, Cleveland, OH) [32]. C20 were maintained in Dulbecco modified Eagle medium (DMEM) supplemented with 10% heat-inactivated fetal bovine serum.

### Production of HIV-1 stocks and infection

pNL4-3 proviral plasmid was obtained through the NIH AIDS Reagent Program, Division of AIDS, NIAID, NIH; from Malcolm Martin. pMD2.G for VSV-G expression was obtained from Jane Burns at UC San Diego. Vesicular stomatitis virus g glycoprotein (VSV-G)-pseudotyped HIV-1 NL4.3 was created by transfection of 293T cells (CRL 3216 from American Type Culture Collection, ATCC) using jetPRIME (Polyplus) transfection reagent according to manufacturer’s instructions. Virus was harvested from transfected cell supernatants at 36 h post-transfection, clarified, filtered through a 0.45-μm filter and stored at − 80 °C. Primary HIV-1 isolate BaL stocks were prepared as follows: Human peripheral blood mononuclear cells (PBMCs) were isolated from fresh heparinized blood by standard Ficoll-Hypaque gradient centrifugation methods. PBMCs were resuspended in RPMI 1640 supplemented with 20% heat-inactivated fetal bovine serum and 50 μg/mL gentamicin (RPMI 1640-GM). Primary HIV-1 isolates were propagated in PBMCs stimulated with 5 μg/mL phytohemagglutinin (PHA) and 5% interleukin 2 (IL-2). The IL-2/PHA-stimulated cells were infected using a high-titer seed stock of virus minimally passaged in PBMCs, starting from a viral stock obtained through the NIH AIDS Reagent Program (from Dr. Suzanne Gartner, Dr. Mikulas Popovic and Dr. Robert Gallo). One mL of virus was transferred to the flask containing freshly stimulated PBMCs and incubated overnight at 37 °C in 5% CO_2_. The cells were washed extensively and resuspended in 30 mL of RPMI-GM with IL-2. Typically, the virus was harvested two times; the first harvest was on day 4 post-infection, with subsequent harvest on day 7. The virus-containing supernatants were collected, clarified by centrifugation, and filtered through a 0.45-μm filter. The virus was then aliquoted into 1-mL sterile screw-cap cryovials and stored at − 80 °C. Infectivity of viral stocks were assayed for infectivity using TZM-bl indicator cells (obtained through the NIH AIDS Reagent Program, Division of AIDS, NIAID, NIH; from Dr. John C. Kappes, Dr. Xiaoyun Wu and Tranzyme Inc.). TZM-bl were incubated for 48 h, and 100 μL of supernatant was removed from each well prior to the addition of 100 μL of Bright Glo substrate (Promega, Madison, WI). Measurement of infectivity involved transfer of 150 μL of cell/substrate mixture to black 96-well solid plates and measurement of luminescence. Cells were infected with primary HIV-1 isolate BaL at an MOI of 0.05, 0.1, 0.25 and 0.5.

### Flow cytometry

Cell surface expression was measured for CD11b-AF488 (Biolegend 101217), CD45-PE (Biolegend 368510), CX3CR1-APC (Biolegend 341610), CD4-PE (Biolegend 368510), CCR5-PE/Cy7 (Biolegend 359108), CXCR4-APC (Biolegend 306510), Siglec-1-APC (Biolegend 346008), CD317-PE (Biolegend 348406) and CD43-APC (Biolegend 343206). Intracellular expression was measured for CD68-APC (Biolegend 333810). Briefly, cells were detached using Versene (Thermo Fisher Scientific) at 4 °C for 15 min with gentle scraping as required, washed and resuspended in PBS. To assess viability, cells were stained with Zombie Violet dye (Biolegend 423113) at 1:500 for 15 min followed by blocking with MACS buffer (Miltenyi Biotec) supplemented with 6 µg/mL human IgG. For CD68 staining, Intracellular CD68 staining was detected using BD Cytofix/CytoPerm kit according to manufacturer’s protocol (BD Biosciences). Conjugated primary antibody immunostaining was performed for 1 h at 4 °C in MACS buffer. Appropriate conjugated isotype antibodies were used as negative controls. Cell-antibody complexes were centrifuged, washed and the pellet resuspended in 300 µl MACS buffer prior to flow cytometric analysis. FACS Canto II and FACSDiva software (BD Biosciences) were used for acquisition and FlowJo software (Treestar) for data analyses. In some experiments, cell death was measured using the Alexa Fluor 488 Annexin V/Dead Cell Apoptosis kit (ThermoFisher Scientific). iPSC-MG1 were infected with primary HIV-1 BaL at an MOI of 0.25. Cells were collected at day 4, 8 and 12 post-infection along with uninfected controls, incubated with Alexa Fluor 488 Annexin V and PI according to the manufacturer’s protocol, and analyzed using a FACS Canto II flow cytometer (BD Biosciences). Data were analyzed using FlowJo v10.6.1 software (BD Life Sciences).

### Immunofluorescence microscopy

MDM and MMG were seeded on type 1 rat tail collagen-coated 35 mm^2^ MatTek dishes and allowed to mature as described. C20 and HMC3 were seeded on poly-d-lysine coated 35 mm^2^ MatTek dishes. Day 28 iMGLs were seeded on fibronectin coated MatTek dishes. At the appropriate time, cells were fixed with 4% paraformaldehyde (PFA) in PBS for 10 min, permeabilized with 0.2% Triton X-100 for 5 min and blocked with Dako serum free protein block (Agilent) supplemented with 6 µg/mL human IgG. Cells were immunostained for P2RY12 (Sigma HPA014518), CX3CR1 (Biorad AHP1589), TMEM119 (Biolegend 853302), IBA-1 (Wako 27030), Siglec-1 (Biolegend 346002), CD9 (BD Pharmingen 555370), Rabbit anti-human tetherin AS [1] and p24-FITC (Beckman Coulter 6604665), in Dako antibody diluent (Agilent), washed several times with PBS supplemented with 0.05% NP-40 and 1% BSA and incubated with the appropriate secondary antibodies. To stain the nucleus, cells were subsequently incubated with 300 nM DAPI (4′,6′-diamidino-2-phenylindole) in PBS for 10 min at room temperature, washed several times with PBS and imaged. Immunofluorescence images were acquired using a DeltaVision RT deconvolution microscope (GE Life Sciences) and data analyses were performed using Volocity Visualization and Quantification Software (Quorum Technologies).

### RNA isolation

Cellular total RNA was isolated from 5 × 10^5^ cells using the RNeasy Mini Kit (Qiagen, 74104). Briefly, cells were pelleted, washed and lysed in RLT buffer prior to centrifugation through a QIAshredder cell-lysate homogenizer (Qiagen, 79654). Samples were further DNase treated according to manufacturer’s instructions. RNA quality control was performed using an Advanced Analytical Technologies, Inc. (AATI) Fragment Analyzer and integrity RIN/RQN values exceeded 9.5. For high throughput RNA sequencing, 450 ng of RNA per sample was used to construct RNAseq libraries employing Illumina TruSeq mRNA standard protocols. Each sample was subsequently sequenced using an Illumina NovaSeq 6000 apparatus.

### Processing of RNA-seq Data and Statistical Analyses

RNA-seq reads in FASTQ format were first subjected to quality control to assess the need for trimming of adapter sequences or bad quality segments. The programs used in these steps were FastQC v0.11.7 [74], Trim Galore! v0.4.2 [75] and cutadapt v1.9.1 [76]. The trimmed reads were aligned to the reference human genome version GRCh38/hg38 with the program STAR v2.6.1e [77]. Aligned reads were stripped of duplicate reads with the program sambamba v0.6.8 [78]. Unnormalized gene expression was assessed by counting features for each gene, as defined in the NCBI’s RefSeq database [79]. Read counting was performed with the program feature Counts v1.6.2 from the Rsubread package [80]. Differential gene expressions between groups of samples were assessed with the R package DESeq 2 v1.26.0 [81, 82]. For heatmaps, we use normalized counts, expressed in transcripts per million (TPM). For measuring similarity of gene expression, the pairwise correlation in expression profiles of all genes (Fig. 4b) or of HIV-relevant genes (Fig. 6b) were calculated. The distribution of correlations coefficients of iPSC-MG vs. AMG (15 correlations) was compared to the distribution of correlations coefficients of HMC3 plus C20 vs. AMG (12 correlations) using the Wilcoxon Rank Sum Test. The same method was used to compare the distribution of correlations coefficients of MMG vs. AMG (6 correlations) with HMC3 plus C20 vs. AMG (12 correlations). Scientific plots were generated using R base graphics as well as with the ggplot2 package [83]. Statistical tests were performed in R using the cor and the wilcox.test function. All datasets generated in this study have been uploaded to the GEO database and are included in Additional file 8: Table S3. Published datasets from the GEO database that we utilized in our analysis are listed in Additional file 9: Table S4.

### Quantitative PCR

RNA was isolated from cell pellets using an RNeasy Mini Kit (Qiagen). The RNA was quantified using a NanoDrop Microvolume Spectrophotometer (ThermoFisher Scientific) and stored at − 80 °C. cDNA synthesis was performed using 1 µg of RNA and the SuperScript IV VILO Master Mix with ezDNase Enzyme (Invitrogen). qPCR amplification reaction was performed using TaqMan probes in a 20 µl reaction in an Applied Biosystems 7500 Fast Real-Time PCR System. The catalog number of the probes were *Gapdh* (Hs99999905_m1), *Cd4* (Hs01058407_m1), *Ccr5* (Hs00152917_m1), *P2ry12* (Hs00375457_m1), *Cx3cr1*(Hs00365842_m1), *Tmem119* (Hs01938722_u1), *Aif1* (Hs00610419_g1), *Siglec1* (Hs00224991_m1), and *Bst2* (Hs00171632_m1). The reactions were performed in triplicate for all genes. Quantification was performed using the comparative C_T_ method using formula 2^−∆∆CT^. Relative fold expression was normalized to levels in MDMs for all genes assayed.

### P24 ELISA

P24 antigen content of HIV-1 BaL viral stocks and infected cell supernatants were measured using a p24 antigen capture ELISA. Murine anti-p24 capture antibody 183-H12-5C (CA183) was obtained from Bruce Chesebro and Kathy Wehrly through the NIH AIDS Research and Reference Reagent Program. Briefly, CA183 hybridoma supernatants were coated onto 96-well plates at a dilution of 1:800 in PBS and incubated overnight at 37 °C. Plates were washed two times with PBS and blocked for 1 h at 37 °C with 5% fetal calf serum (FCS) in PBS. Samples measured were diluted in p24 ELISA sample diluent containing 5% FCS and 0.5% Triton X-100 in PBS and incubated for 2 h at 37 °C. Plates were then washed four times with 0.1% Tween 20 in PBS. The detection of bound p24 was determined using HIV-Ig, obtained from NABI through the NIH AIDS Research and Reference Reagent Program, at a dilution of 1:20,000 in p24 ELISA sample diluent for 1 h at 37 °C. Plates were then washed four times with 0.1% Tween 20 in PBS and incubated with goat anti-Human IgG (H + L) Cross-Adsorbed Secondary Antibody, HRP (ThermoFisher, 31412) at a dilution of 1:5000 in p24 ELISA sample diluent. Plates were washed four times with 0.1% Tween 20 in PBS and colorimetric analysis was performed using the Immunopure TMB Substrate Kit (Pierce, Rockford, IL). From 10 to 30 min later reactions were stopped with 4 N H2SO4 and absorbance read at 450 nm. Recombinant p24 was used for the standard curve and sensitive to less than 20 pg of p24.

## Supplementary information

**Additional file 1: Fig. S1.** iPSC72.3 were differentiated into non-adherent hematopoietic progenitor cells (HPCs) and assayed for cell surface expression of CD43 by flow cytometry. Non-adherent cells are greater than 98% CD43+. Isotype controls are shown in red; CD43+ staining in blue.

**Additional file 2: Table S1.** Table of 780 microglia-enriched genes as outlined in the text, related to Fig. 4b.

**Additional file 3: Fig. S2.** Hierarchal clustering of primary human microglia and model systems. Correlation matrix of 780 microglia-enriched genes from Fig. 4b. (R, Pearson’s correlation coefficient). Analysis performed using log2-transformed TPM values of 780 transcripts enriched in primary human adult microglia.

**Additional file 4: Table S2.** Listing of fifteen selected microglial genes relevant to Fig. 5.

**Additional file 5: Fig. S3.** qPCR validation of microglial-enriched and HIV-associated gene expression. Levels were normalized to GAPDH and expression presented as relative to MDM. Data are presented as mean ± SD. *P2ry12*, purinergic receptor P2Y12, *Tmem119*, transmembrane protein 119, *Cx3cr1*, C-X3-C motif chemokine receptor 1, *Cd4*, CD4 receptor, *Ccr5*, C–C motif chemokine receptor 5, *Aif1*, allograft inflammatory factor 1, *Siglec1*, sialic acid binding Ig like lectin 1, *Bst2*, bone marrow stromal cell antigen 2/tetherin.

**Additional file 6: Fig. S4.** HIV replication kinetics in transformed microglia cell lines C20 and HMC3. Cells were cultured and infected at a MOI of 0.25 with either (A) primary HIV-1 isolate HIV-1_BaL_ or (B) VSV-G-pseudotyped NL4.3. Culture supernatants from HIV-infected C20 and HMC3 were collected at days 0, 2, 4, 6, 8, 10, 12 and 14 post-infection and analyzed for p24 expression by antigen capture ELISA.

**Additional file 7: Fig. S5.** Similarity of HIV replication kinetics between iPSC-MG1 and MG-CD at different MOI. iPSC-MG1 and MG-CD were infected with a biological stock of HIV-1_BaL_ at indicated MOIs. Cell culture supernatants were collected at days 0, 2, 4, 6, 8, 10, 12 and 14 post-infection and p24 measured by antigen capture ELISA.

**Additional file 8: Table S3.** RNAseq datasets included in the analysis of this paper and submitted to the GEO database.

**Additional file 9: Table S4.** Listing of published datasets downloaded from the GEO database and used as comparators in this study.

## Data Availability

The datasets obtained during the current study are available in GEO (NCBI), including metadata spreadsheets, processed data files, and raw data files. GEO Accession numbers listed in Additional file 8: Table S3, and the TPM and read counts included in Additional file 9: Table S4.
