# Supplementary figures and images for: Comparative analysis of human microglial models for studies of HIV replication and pathogenesis
Source: Retrovirology. 2020 Nov 19;17:35. doi: 10.1186/s12977-020-00544-y (PMC7678224; doi:10.1186/s12977-020-00544-y)

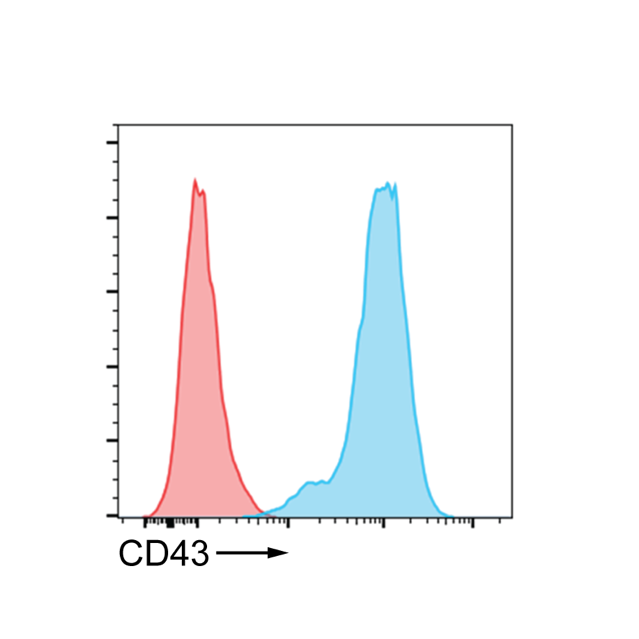

Supplement: Supplementary file 1 — Additional file 1: Fig. S1. iPSC72.3 were differentiated into non-adherent hematopoietic progenitor cells (HPCs) and assayed for cell surface expression of CD43 by flow cytometry. Non-adherent cells are greater than 98% CD43+. Isotype controls are shown in red; CD43+ staining in blue. [file 12977_2020_544_MOESM1_ESM.jpeg]

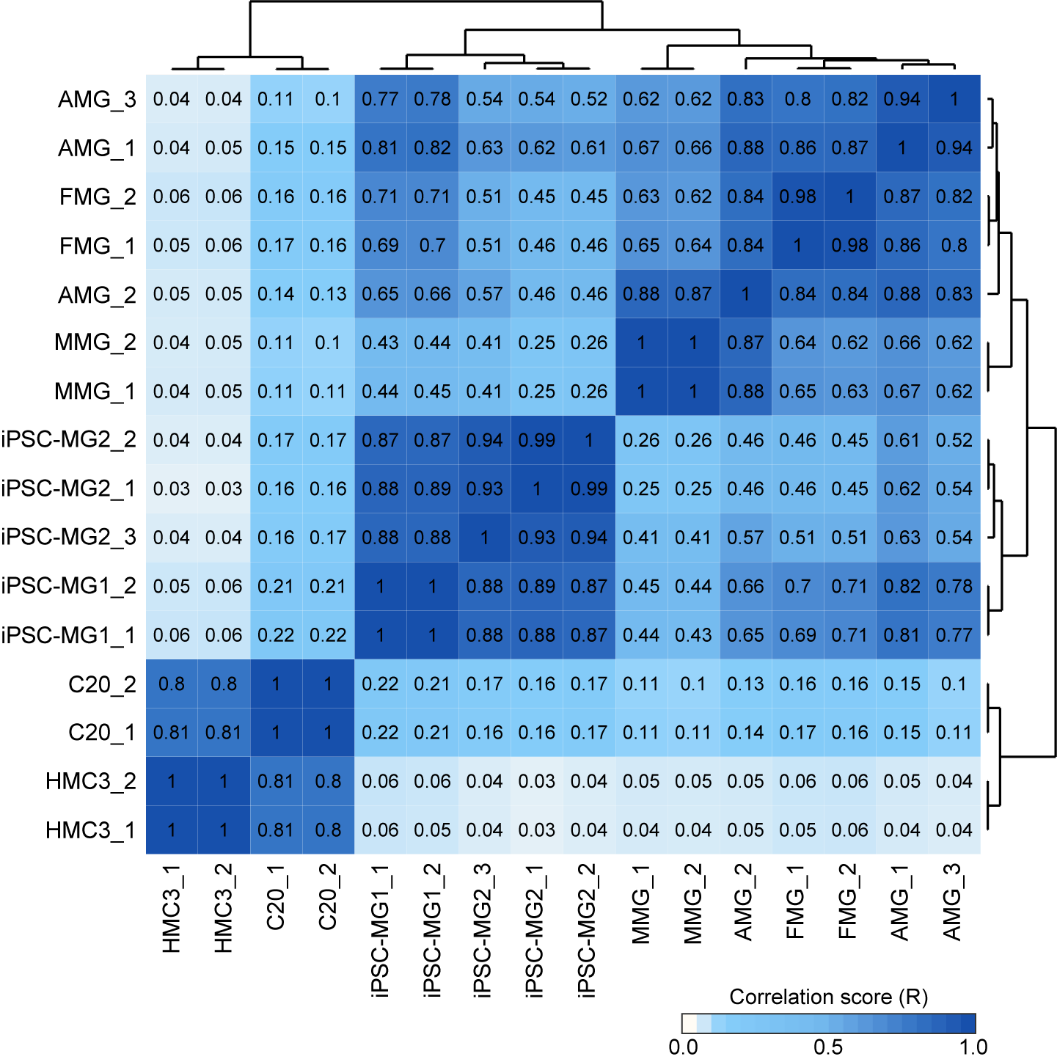

Supplement: Supplementary file 3 — Additional file 3: Fig. S2. Hierarchal clustering of primary human microglia and model systems. Correlation matrix of 780 microglia-enriched genes from Fig. 4b. (R, Pearson’s correlation coefficient). Analysis performed using log2-transformed TPM values of 780 transcripts enriched in primary human adult microglia. [file 12977_2020_544_MOESM3_ESM.jpeg]

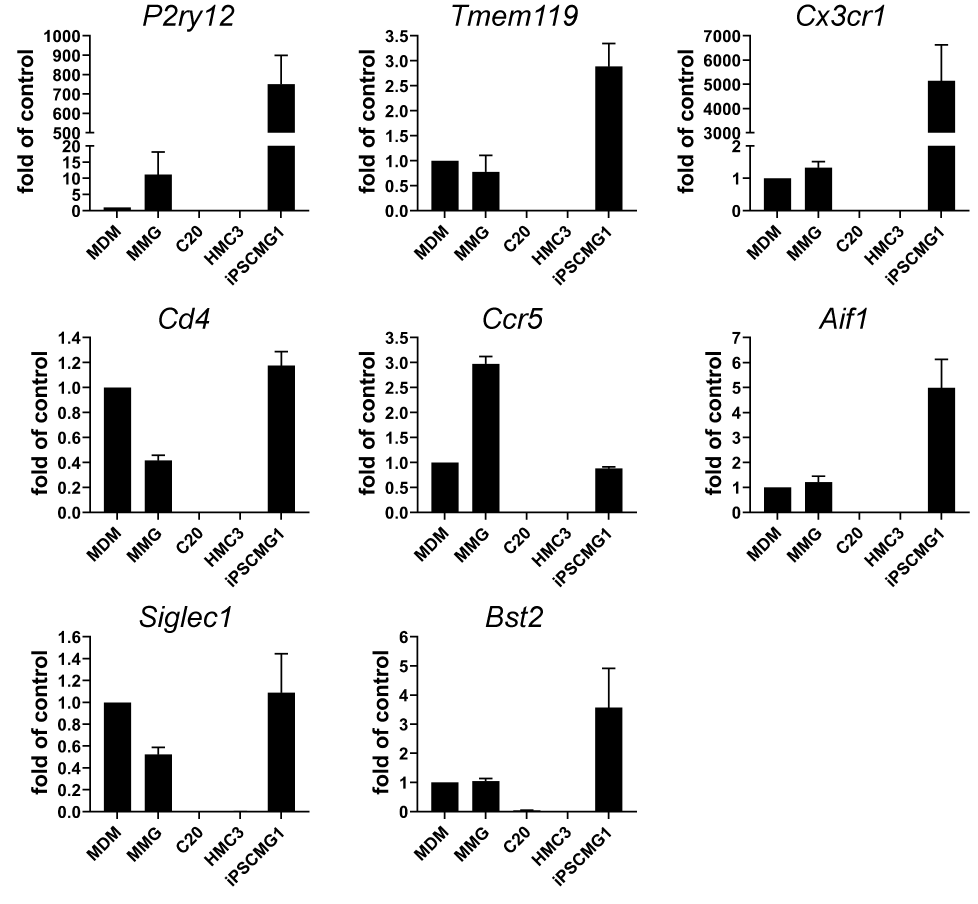

Supplement: Supplementary file 5 — Additional file 5: Fig. S3. qPCR validation of microglial-enriched and HIV-associated gene expression. Levels were normalized to GAPDH and expression presented as relative to MDM. Data are presented as mean ± SD. P2ry12, purinergic receptor P2Y12, Tmem119, transmembrane protein 119, Cx3cr1, C-X3-C motif chemokine receptor 1, Cd4, CD4 receptor, Ccr5, C–C motif chemokine receptor 5, Aif1, allograft inflammatory factor 1, Siglec1, sialic acid binding Ig like lectin 1, Bst2, bone marrow stromal cell antigen 2/tetherin. [file 12977_2020_544_MOESM5_ESM.jpeg]

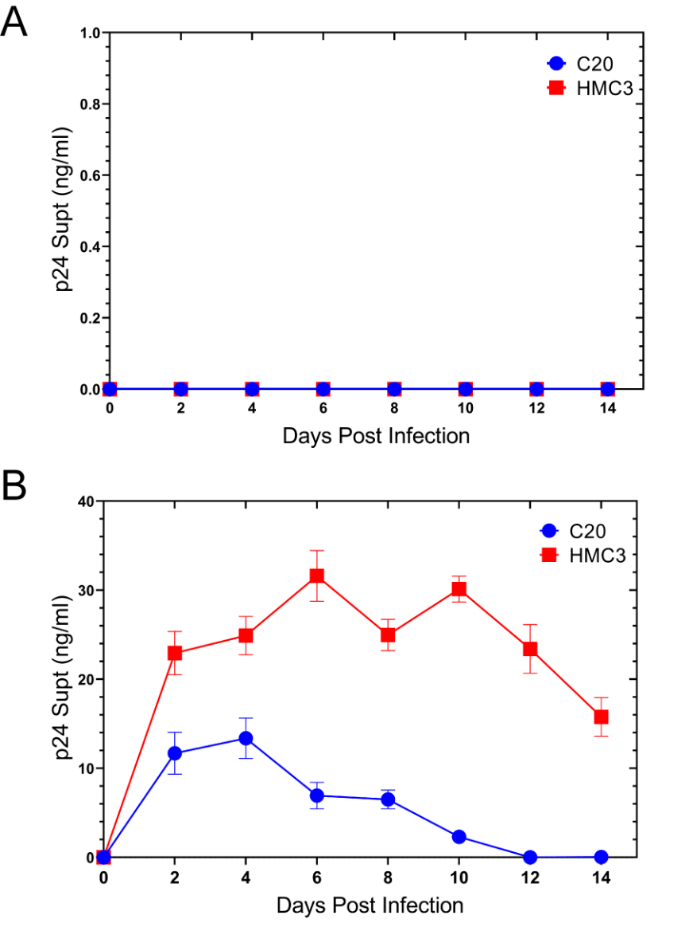

Supplement: Supplementary file 6 — Additional file 6: Fig. S4. HIV replication kinetics in transformed microglia cell lines C20 and HMC3. Cells were cultured and infected at a MOI of 0.25 with either (A) primary HIV-1 isolate HIV-1BaL or (B) VSV-G-pseudotyped NL4.3. Culture supernatants from HIV-infected C20 and HMC3 were collected at days 0, 2, 4, 6, 8, 10, 12 and 14 post-infection and analyzed for p24 expression by antigen capture ELISA. [file 12977_2020_544_MOESM6_ESM.jpeg]

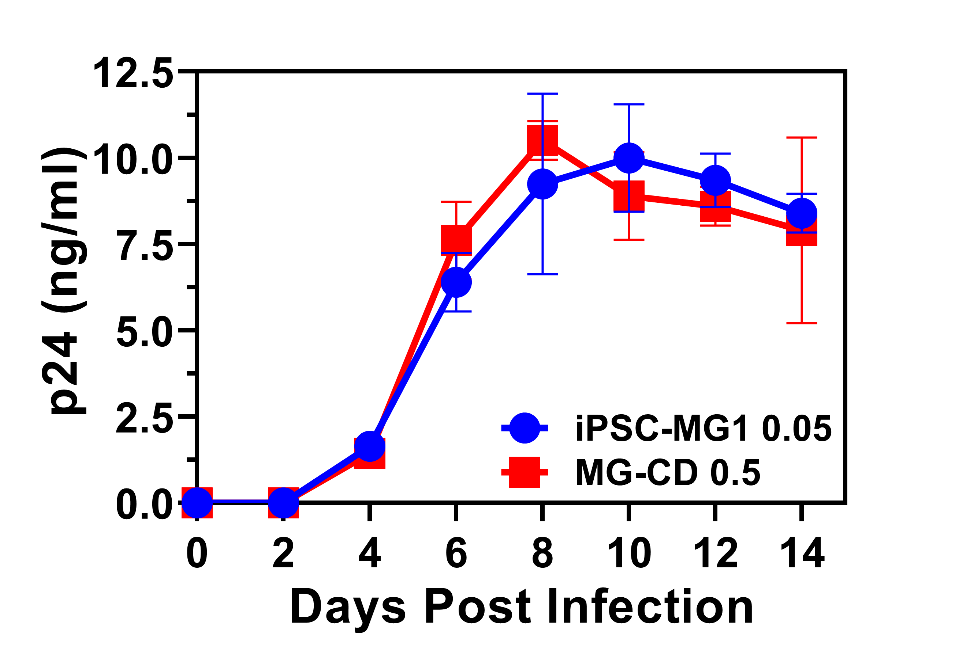

Supplement: Supplementary file 7 — Additional file 7: Fig. S5. Similarity of HIV replication kinetics between iPSC-MG1 and MG-CD at different MOI. iPSC-MG1 and MG-CD were infected with a biological stock of HIV-1BaL at indicated MOIs. Cell culture supernatants were collected at days 0, 2, 4, 6, 8, 10, 12 and 14 post-infection and p24 measured by antigen capture ELISA. [file 12977_2020_544_MOESM7_ESM.jpeg]
